# Circulating tumor DNA as part of the routine work-up for patients with suspected advanced lung cancer^☆^

**DOI:** 10.1016/j.jlb.2025.100443

**Published:** 2025-11-05

**Authors:** Emma Holjak, Tia Brasoveanu, Saurav Verma, Saqib Khan, Morgan Black, Inderdeep Dhaliwal, Michael Mitchell, Rahul Nayak, Mehdi Qiabi, Richard Inculet, Dalilah Fortin, Richard Malthaner, Matthew Cecchini, Anna Lapuk, Daniel Breadner

**Affiliations:** a Schulich School of Medicine and Dentistry, London, Ontario, Canada; b Division of Medical Oncology, Verspeeten Family Cancer Centre, London, Ontario, Canada; c Department of Oncology, Schulich School of Medicine and Dentistry, London, Ontario, Canada; d Division of Respirology, Department of Medicine, Western University, London, Ontario, Canada; e Division of Thoracic Surgery, Western University, London, Ontario, Canada; f Department of Surgery, Schulich School of Medicine and Dentistry, London, Ontario, Canada; g Division of Surgical Oncology, Department of Oncology, Schulich School of Medicine and Dentistry, London, Ontario, Canada; h Department of Pathology and Laboratory Medicine, London, Ontario, Canada; i Imagia Canexia Health, Vancouver, BC, Canada; j Avitia Inc, Montreal, QC, Canada

**Keywords:** Liquid biopsy, NSCLC, ctDNA

## Abstract

Liquid biopsy (LB) is a useful tool in patients with advanced non-small cell lung cancer (aNSCLC) to detect actionable molecular alterations and thereby allow genotype-matched therapies. Currently, LB is recommended for individuals diagnosed with aNSCLC who have an insufficient tissue sample or difficult-to-reach tumour tissue. Despite the potential advantages of LB, its incorporation into the standard diagnostic work-up for all newly diagnosed patients with aNSCLC is lacking. Our study aimed to evaluate whether addition of plasma circulating tumor DNA (ctDNA) next generation sequencing (NGS) testing early in the diagnostic work-up for patients with aNSCLC can improve the time to molecular results and treatment initiation. This was a single-centre quality improvement initiative with two cohorts of patients. Patients in the ‘ctDNA cohort’ had plasma ctDNA testing in addition to the standard diagnostic work-up. The ‘reference cohort' was a parallel group of patients who had the standard work-up (no LB). Tissue biopsy and reflex tissue NGS testing were done in both cohorts. ctDNA testing shortened the time to molecular results in the ctDNA cohort compared to the reference cohort (median, 14 vs 35 days; p = 0.01), the time from first respirology/thoracic surgery consult to molecular results (median, 22 vs 48 days respectively; p = 0.01), and the time from medical oncology consultation to initiation of first-line treatment (median, 12 vs 22 days; p = 0.01). In conclusion, in a publicly funded and single-payer healthcare system, ctDNA testing as part of the standard work-up for patients with aNSCLC provides molecular results significantly faster than tissue-based testing and shortens the time to treatment initiation.

## 1 Introduction

Lung cancer is the most common type of cancer and the leading cause of cancer-related deaths globally [1]. A better understanding of the molecular landscape of non-small cell lung cancer (NSCLC) coupled with advancements in systemic treatment such as targeted therapies and immune checkpoint inhibitors (ICIs) has paved the way for personalized, genotype-matched treatments. This progress has led to significant improvements in survival and quality of life for patients with advanced NSCLC (aNSCLC) [2]. For a patient with aNSCLC, particularly advanced non-squamous NSCLC (aNSq NSCLC), the choice of optimal first-line (1L) treatment is guided by genomic biomarkers identified through comprehensive genomic profiling (CGP). This genomic information from CGP is vital for developing an effective treatment strategy, as it enables the identification of actionable genomic alterations (AGAs) to determine eligibility for targeted therapies. The number of AGA is continuing to grow in first and second-line indications, and now includes EGFR, ALK, NTRK, RET, MET, ROS1, KRAS G12C, ERBB2/HER2, BRAF V600E, and NRG1 [2]. The absence of genotyping results before starting 1L therapy has been linked to inferior survival [3]. As per the current standard of care (SoC), the CGP is usually done by next-generation sequencing (NGS) following a pathological diagnosis of NSq NSCLC, which prompts reflex genotyping on tumor tissue to identify AGAs [4].

The American Society of Clinical Oncology, College of American Pathologists, International Association for the Study of Lung Cancer, and Association for Molecular Pathology recommend a turnaround time (TAT) of 10 business days or less from the tissue sample being received to the reporting of results on AGAs [5,6]. The process from invasive tissue biopsy to pathological diagnosis and molecular results can take anywhere from a few days to many weeks, depending on the system and level of funding [[7], [8], [9]]. More specifically, in recent publications focusing on aNSCLC, TAT for tissue-based NGS has been reported at 32.5 days at a regional cancer centre affiliated with a large community hospital in Canada [9], 27 days at a large community hospital in USA [8], and 18 days for patients within the US Oncology Network database [7]. Timely TAT remains an obstacle in optimal patient care and is influenced by a variety of factors, including delays in: sample acquisition, specimen transportation, sample analytics, and reporting of results.

Plasma-based ctDNA testing, a type of liquid biopsy (LB), is a non-invasive and reliable method for genotyping in newly diagnosed patients with NSCLC and its results complement tissue-based analysis [10,11]. Several studies have shown that LB generally offers quicker TAT compared to CGP on tumor tissue [12,13]. However, despite its potential advantages, LB has not become a standard part of initial work-up for all newly diagnosed patients with aNSCLC. A negative LB result does not rule out AGAs, and tissue-based CGP is still needed in such cases. At present, it is primarily used for patients with insufficient tissue samples or tumors that are difficult to access.

Concurrent plasma-based ctDNA testing, alongside SoC pathology and tissue-based genotyping, has the potential to provide faster information on AGAs. This approach can lead to faster treatment initiation in these patients. This study was done to determine whether early plasma-based ctDNA testing during the diagnostic work-up for patients with aNSCLC can shorten the time to molecular results and/or the start of 1L systemic treatment.

## 2 Methods

This ctDNA diagnostic assessment pathway (ctDNA DAP) study was a prospective, single-centre, investigator-initiated, two-cohort, quality improvement study. The study was conducted at Verspeeten Family Cancer Centre, London, Ontario, Canada. Patients with newly diagnosed aNSCLC were enrolled between March 14th, 2022, and August 11th, 2023. The study was approved by the University of Western Ontario's Health Sciences Research Ethics Board (123139) and all prospectively enrolled patients provided informed consent. The study was carried out in accordance with the principles of Declaration of Helsinki [14].

Eligible patients were 18 years of age or older and had aNSCLC. Patients with a history of other malignancies in the last five years (except adequately treated non-melanoma skin cancer), previous lung cancer biopsy, or previous surgery with biomarker testing were excluded. In the ‘ctDNA cohort’, patients had plasma-based ctDNA NGS testing in addition to the SoC diagnostic work-up. The ‘reference cohort’ included patients who had the SoC work-up with tissue-based NGS. Diagnostic tissue biopsy and reflex tissue-based NGS were done in both cohorts. NGS testing of ctDNA samples was done using Follow It® gene panel (Imagia Canexia Health, Vancouver, BC, Canada) through the Project Access to Cancer Testing and Treatment (supported by the Canadian Technology Digital Supercluster). Follow It® is a targeted amplicon-based assay validated in CAP/CLIA setting to detect somatic alterations in DNA at >337 hotspots and 26 exons in 38 cancer-associated genes simultaneously, including key lung cancer genes such as EGFR, KRAS, BRAF, ERBB2, MET, RET, ALK and others. Detected ctDNA aberrations included single-nucleotide variants (SNVs), insertions and deletions (up to 24bp) and copy number changes of select genes. Tissue NGS was performed using the Ion AmpliSeq™ technology on (ThermoFisher). Library preparation and was performed as per manufacturer’s instructions using the Ion Torrent™ Oncomine™ Comprehensive Assay v3 (DNA) and the Ion Torrent™ Oncomine™ Comprehensive Assay Plus (RNA), both from ThermoFisher. Together, this technique screens 135 cancer driver genes (87 hotspot genes and 48 genes with full exon coverage) and 49 fusion driver genes. 14 DNA genes and 14 fusion genes were reported to the clinician.

This study aimed to evaluate whether plasma-based ctDNA testing early in the diagnostic work-up of patients with aNSCLC improved the time to molecular results and/or time to 1L treatment initiation. Several different outcome measures were assessed. For the ctDNA group, the time from blood draw to molecular results was calculated. Typically, patients would receive a requisition for blood draw at their initial cancer appointment. For both groups, the time from tissue biopsy to molecular results (ctDNA or tissue-based NGS, whichever was reported earlier) was calculated and compared. Similarly, the time from the first respirology/thoracic surgery consult to molecular results was calculated and compared. Lastly, the time from medical oncology consultation to initiation of a 1L treatment was calculated and compared.

### 2.1 Data analysis

Data was extracted from patient medical records. Baseline characteristics were summarized using descriptive statistics. All categorical variables were reported as frequency counts and proportions. All continuous variables were reported as medians with interquartile ranges as appropriate. Comparisons of outcome measures were conducted using non-parametric Wilcoxon-Mann-Whitney test and the Wilcoxon signed rank test. All statistical tests were two-sided, and the significance level was defined *a priori* as < 0.05. Data entry was done on REDCap and imported into Microsoft Excel(R). Statistical analyses were performed using Stata (StataCorp. 2013. Stata Statistical Software: Release 13. College Station, TX: StataCorp LP).

## 3 Results

**Fig. 1:**
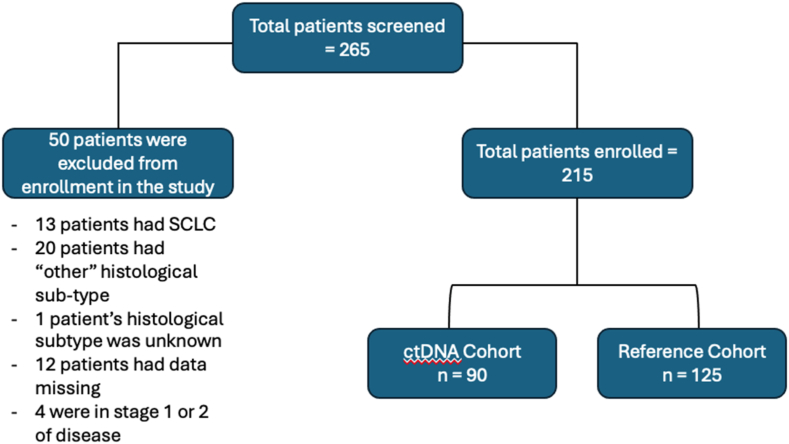
Study flow Diagram.

Data was analyzed from 215 patients (Fig. 1), with 90 patients in the ctDNA cohort and 125 patients in the reference cohort. Both cohorts were similar in terms of age, sex and histological distribution. The median age was 71 years (range, 35–89) in the ctDNA cohort and 72 years (range, 47–89) in the reference cohort. In the ctDNA cohort, there were 39 (43 %) males and 51 (57 %) females. In the reference cohort, there were 72 (58 %) males and 53 (42 %) females. The demographic and disease characteristics are shown in Table 1. Within both cohorts, the most common histological subtype was adenocarcinoma, followed by squamous cell carcinoma, and lastly NSCLC not otherwise specified (NOS).

**Table 1:** Demographic characteristics of the ctDNA and reference cohorts.

Characteristic	ctDNA cohort (No. (%) of patients, N = 90)	Reference cohort (No. (%) of patients, N = 125)
Age, years	71	72
**Sex**		
Male	39 (43)	72 (58)
Female	51 (57)	53 (42)
**Histology**		
Adenocarcinoma	60 (67)	78 (62)
Squamous cell carcinoma	23 (26)	31 (25)
NSCLC NOS	7 (7)	16 (13)
**Clinical stage**		
4	65 (72)	125 (100)
3	25 (28)	0 (0)

No., number; NSCLC NOS, non-small cell lung cancer not otherwise specified.

**Fig. 2:**
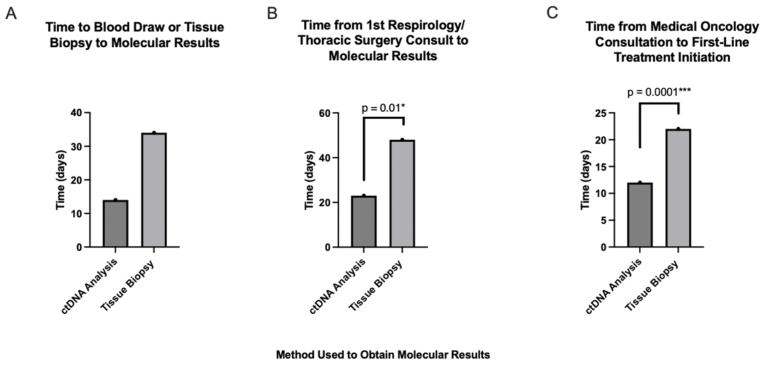
**Graphical depiction of key outcome measures.** A) Median time from blood draw to ctDNA results compared to median time from tissue biopsy to molecular results from tissue NGS. B) Median time from first respirology/thoracic surgery consult to molecular results. C) Median time from first medical oncology consultation to 1L initiation.

The various time parameters for the ctDNA and reference cohorts are shown in Table 2. The molecular results from ctDNA NGS were available significantly faster than the molecular results from the SoC tissue-based NGS testing. The median time from blood draw to molecular results in the ctDNA cohort was 14 days, compared to 35 days for tissue biopsy to molecular results in the tissue-based NGS cohort (p = 0.01; Fig. 2A). As expected, the median time from tissue biopsy to molecular results from tissue-based NGS was similar between groups (34 days and 35 days in the ctDNA and reference cohorts, respectively; p = 0.28). The median time from first respirology/thoracic surgery consult to molecular results from ctDNA NGS was 22 days, whereas the median time from first respirology/thoracic surgery consult to tissue-based NGS results was 48 days (p = 0.01; Fig. 2B). As anticipated, between the ctDNA and reference cohort, the median time from first respirology/thoracic surgery consult to molecular results from tissue biopsy was similar (46 days vs 51 days, p = 0.34). For both groups, the time from first respirology/thoracic surgery consult to tissue biopsy was similar (9 days vs 16 days, p = 0.30).

**Table 2:** Turnaround times for the ctDNA and reference cohorts. IQR, interquartile range; NA, not applicable; 1L, first line.

Time (days)	ctDNA cohort	Reference cohort	P-value
	median (IQR)		
First respiratory/thoracic surgery consult to tissue biopsy	9 (6–35)	16 (6–38)	0.30
First respiratory/thoracic surgery consult to ctDNA results	22 (18–38)	NA	NA
First respiratory/thoracic surgery consult to tissue NGS results	46 (37–60)	51 (37–74)	0.34
First respiratory/thoracic surgery consult to initiation of 1L systemic therapy	56 (44–71)	64 (47–105)	0.10
Blood draw to ctDNA results	14 (12–15)	NA	NA
Tissue biopsy to date of initial tissue diagnosis reported	7 (6–9)	7 (6–10)	0.33
Tissue biopsy to date of tissue NGS results	34 (30–40)	35 (32–41)	0.28
Tissue biopsy to initiation of 1L systemic therapy	41 (30–55)	57 (44–87)	0.10
First medical oncology appointment to initiation of 1L systemic therapy	12 (6–22)	22 (10–44)	0.01

To assess whether ctDNA testing improved time to 1L treatment initiation, several different timeframes were compared between the cohorts. The time from tissue biopsy to start of 1L systemic therapy was not significantly different between the ctDNA and reference cohorts (41 days in the ctDNA cohort vs 57 days in the reference cohort, p = 0.10). Similarly, there was no significant reduction in time from first respiratory/thoracic surgery consult to start date of 1L systemic therapy in the ctDNA cohort vs the reference cohort (56 days and 64 days respectively, p = 0.10). Notably, the time from medical oncology consultation to 1L treatment initiation was shorter in the ctDNA cohort compared to the reference cohort (12 vs 22 days, respectively; p = 0.01; Fig. 2C). In summary, ctDNA testing shortened the time from the patient’s first medical oncology appointment to treatment initiation, but not the time from first respirology/thoracic surgery consult to treatment initiation.

Within the group that had both tissue NGS and ctDNA testing, ctDNA alone would result in fewer patients having an AGA identified (30 vs 4 patients with no gene found on ctDNA vs tissue-based analysis) (Table 3). Between these two methods, there were differences in the AGA identified within one patient. In patients that had ctDNA analysis, there were instances where a AGA was only identified through ctDNA analysis but not tissue NGS (Ex. NRAS, EGFR, KRAS, etc.). Similarly, there were also AGAs identified through tissue NGS that were not found on ctDNA analysis with one patient (Ex. FGFR1, ALK, ROS1, etc.).

**Table 3:** Molecular Results from Tissue NGS and ctDNA testing.

Cohort 1 – Tissue NGS and ctDNA NGS																		
Gene	**ALK**	**BRAF**	**EGFR**	**ERBB2**	**FGFR1**	**KRAS**	**MET**	**NRAS**	**NTRK1**	**NTRK3**	**PIK3CA**	**RET**	**ROS1**	**SMARCA4**	**TP53**	**5**	**3**	**None**
Tissue	3	5	11	1	1	18	2	0	0	0	1	0	1	2	38	6	4	4
ctDNA	1	2	13	1	0	15	9	2	0	0	3	0	0	0	37	6	1	30

## 4 Discussion

The timely availability of genotyping results, particularly the information on AGAs, as well as the PD-L1 tumour proportion score (TPS), are critical for initiating an effective treatment in patients with newly diagnosed aNSCLC. For patients with specific AGAs (e.g., EGFR mutations, ALK/ROS1/RET fusions), 1L targeted therapy is recommended. Otherwise, immunotherapy with or without chemotherapy is typically used. However, delay in obtaining tissue-based NGS results due to factors like inadequate or unavailability of tissue and long turnaround time (TAT), can delay treatment initiation and potentially worsen patient outcomes.

This prospective study aimed to evaluate whether plasma-based ctDNA testing improved time to molecular results and 1L treatment initiation in patients with aNSCLC. The study showed that 1L treatment can be initiated 45 % faster using ctDNA analysis (i.e., median time from medical oncology consultation to initiation of 1L treatment, 12 days vs 22 days). This difference was largely attributed to the earlier availability of molecular results with plasma-based NGS, as the median TAT for plasma-based NGS was 14 days compared to 34 days for tissue-based NGS.

The debate between the "tissue-first" and "plasma-first" approaches remains ongoing [15]. Several studies have explored the "plasma-first" approach, where liquid biopsy is used initially, followed by tissue NGS if liquid biopsy results are negative. For instance, during the COVID pandemic, Cui et al. showed that 22 % of patients started targeted therapy based solely on cfDNA (circulating free DNA)-NGS results, without tissue genotyping results [16]. Another study showed that plasma-based genotyping before pathological diagnosis in hospitalized patients led to substantially shorter TAT for genotyping compared to standard outpatient workflows [17]. A study in China screened 391 patients with lung cancer without available tissue for EGFR alterations using LB. Of those, 116 were found positive for the typical EGFR mutations leading to initiation of targeted therapy, illustrating the clinical value of early LB [18]. The "plasma-first" approach also avoids tissue exhaustion when the tissue is scant, and this valuable tissue may then be available for immunohistochemistry for calculating PD-L1 TPS, as well as, for upcoming targets such as TROP-2. Reflex tissue-based testing is still recommended in cases where plasma results are negative [10].

Increasing evidence supports a complementary strategy, where both plasma and tissue-based testing are performed simultaneously to ensure a more comprehensive evaluation [3,10,19]. The IASLC consensus statement endorses LB as a valid tool for genotyping in aNSCLC, recognizing its complementary role to tissue-based NGS. Multiple studies suggest that the concurrent approach with plasma and tissue-based genotyping is better than either alone. In NSCLC patients who had a LB in addition to tissue NGS, there was a reduction in time to molecular results, a reduction in time to treatment initiation, faster TAT, and an increase in the detection of AGAs [12,[20], [21], [22], [23]]. In fact, when both tissue-based NGS and LB are used to detect AGAs, it has been found that the combination of both methods is superior to either method alone [12,21]. The recently reported PLAN study also demonstrated that LB at the time of radiologic suspicion reduced time to genomic diagnosis by three weeks, had 90 % concordance with the variants found in tissue, and showed cost savings in micro-cost analysis [24].

Despite the advantages of plasma-based genotyping, including faster TAT compared to tissue-based genotyping, its incorporation into routine clinical care is lacking [15]. When tumor burden is high, the high negative predictive value of ctDNA suggests confidence in negative results, allowing prioritization of reflex-tissue testing for cases with low ctDNA levels [25]. However, in cases with low tumor burden plasma-based testing may yield a false negative, highlighting the need for confirmatory tissue-based genotyping. Therefore, tissue biopsy remains SoC and essential after pathologic confirmation and subtyping, especially if plasma NGS is negative. Also, non-DNA biomarkers (e.g., RNA or protein markers) are not usually evaluable through LB.

The cost-effectiveness of the concurrent approach remains an important consideration. Eziefe and colleagues assessed the cost-effectiveness of adding LB to tissue-based testing for the detection of AGAs in aNSCLC in the Canadian healthcare system (i.e., single payer). The study showed that the addition of LB to tissue-based testing can identify AGAs in more patients (68.5 %) compared to tissue-based testing alone (52.7 %). This approach led to incremental cost savings and a small gain of quality-adjusted life-years, as it allowed more patients to receive appropriate targeted therapy [26]. Furthermore, a recent paper published by Scheffield and colleagues investigated how implementing a rapid in-house LB service affected the treatment course in a community healthcare center in Canada [27]. Their rapid in-house LB system had a median turnaround time of 3 days. As discussed by the authors, this expedited turnaround time not only reduced time to treatment for patients but also minimized the typical cost ($400 per patient per delayed week of care) associated with providing care to these patients in the interim while awaiting investigation results [27,28]. Despite aNSCLC being a heterogeneous disease, certain mutations can be very responsive to therapy. In these cases, early access to targeted therapy can prevent hospitalization and shorten hospital stays. Ultimately, while adding a concurrent investigation (i.e., LB) into the current diagnostic workup for aNSCLC patients initially would present as an added cost, the potential savings gained by expediting the diagnostic results and reducing the interim care costs seem to help offset the cost of this additional test. Although here we demonstrated significant (>two-fold) improvement in time to molecular result using ctDNA profiling, it is worth noting that the current study relied on centralized testing using Canexia Health laboratory (Vancouver, BC). Local deployment of such a solution would further reduce TAT compared to tissue-based methods, leading to greater cost and time savings for patients and local healthcare centers [29,30].

An additional benefit of using LB in the diagnostic workup of aNSCLC is the quality of life improvements that this approach affords patients. Our data shows that LB facilitates a quicker turnaround time to diagnosis and earlier treatment initiation. Together, this can result in earlier symptom relief for patients and thus improve their quality of life. Using LB in addition to the standard tissue-based NGS workup allows for more targetable alterations to be found than either approach alone [12,21]. Ultimately, this allows for more patients to receive first-line targeted therapy, which has been shown to improve symptom management [31] and improve survival [3].

The limitation of our study includes its single-centre, non-randomized design and a comparison to a reference cohort. Additionally, the DNA-only targeted gene panel covered genomic alterations in 38 key genes, except fusions, although all eligible patients underwent SoC tissue-based (DNA genes and RNA fusions genes) NGS testing for all approved targetable alterations, including fusions. This emphasizes the need for a cost-effective LB platform for detecting RNA-based fusions in blood. Additionally, our study neither included a cost-effectiveness nor a cost-utility analysis.

In conclusion, this study demonstrates that in a publicly funded and single-payer healthcare system, plasma-based ctDNA testing as part of the standard diagnostic work-up for patients with aNSCLC can provide molecular results significantly faster compared to the traditional tissue-based testing alone. This approach also accelerated the treatment initiation by as much as 45 %. The findings, along with other supporting studies, suggest that LB is becoming an increasingly valuable and complementary tool in the genotyping of patients with aNSCLC. The integration of LB into the routine diagnostic process and clinical practice could potentially improve treatment initiation and enhance patient outcomes. However, further studies are needed to address the challenges of low tumor burden and the cost-effectiveness and cost-utility of this approach in broader healthcare settings. The integration of LB into routine clinical practice faces regulatory and reimbursement hurdles in different health systems.

## Statement of ethical approval

The study was approved by the University of Western Ontario's Health Sciences Research Ethics Board (123139) and all prospectively enrolled patients provided informed consent.

## Declaration of competing interest

The authors declare the following financial interests/personal relationships which may be considered as potential competing interests:Daniel Breadner reports financial support was provided by AstraZeneca Canada Inc. This work was conducted while co-author Anna Lapuk was employed at Imagia Canexia Health, the assets of which have been acquired by Avitia Inc. If there are other authors, they declare that they have no known competing financial interests or personal relationships that could have appeared to influence the work reported in this paper.
